# Genome Sequence of Lactiplantibacillus plantarum DmPark25_157, a Bacterial Strain Isolated from Drosophila melanogaster

**DOI:** 10.1128/MRA.01372-20

**Published:** 2021-04-22

**Authors:** Hunter H. Giles, Samara C. Petersen, Gerald B. Call, John M. Chaston

**Affiliations:** a Department of Plant and Wildlife Sciences, Brigham Young University, Provo, Utah, USA; b Department of Pharmacology, Midwestern University, Glendale, Arizona, USA; University of Maryland School of Medicine

## Abstract

We present the genome sequence of a bacterial strain isolated from *park^25^* mutants of Drosophila melanogaster as part of efforts to better understand the microbial communities in D. melanogaster. We isolated and sequenced a Lactiplantibacillus plantarum strain. We present a preliminary comparative analysis with a closely related strain.

## ANNOUNCEMENT

Lactiplantibacillus plantarum isolates are of interest in food industry fermentation (1) and are commonly found in gut microbiomes (2). Strains have recently been investigated as a possible protection against intestinal inflammation (3). We are building a genomic resource for potential microbial interactions with Parkinson’s disease.

D. melanogaster with the *park^25^* mutation has been used as a model of Parkinson’s disease (4). We used a laboratory stock reared on a Bloomington-style diet, with the following components per liter: 957 ml water, 11.8 g yeast, 5.9 g soy flour, 58.8 g yellow cornmeal, 35.3 ml light corn syrup, 35.3 ml molasses, 4.4 ml propionic acid, and 10 ml 10% methylparaben in 95% ethanol. We took a stock of the *park^25^* mutant from standard rearing conditions (25°C, 12/12-h light/dark cycle, and ambient humidity) in a Glendale, AZ, laboratory and homogenized 5 whole-body flies in phosphate-buffered saline (PBS); then, we dilution plated the homogenate onto modified MRS medium (5) and incubated the cultures at 30°C in an airtight CO_2_-flooded container for 48 h. We streaked large, flat, or wrinkled yellow colonies for isolation and sequenced portions of 16S rRNA by Sanger sequencing, using universal 27F and 1492R primers (27F, 5′-AGAGTTTGATCMTGGCTCAG-3′; 1492R, 5’-TACGGYTACTACCTTGTTACTT-3’). We selected one of these strains for whole-genome sequencing.

We extracted DNA from the bacterial colony using the Qiagen DNeasy PowerLyzer microbial kit. The DNA was fragmented to 500 bp using NEBNext double-stranded DNA (dsDNA) Fragmentase (M0348L) and end repaired using the NEBNext Ultra II end repair/dA-tailing module (E7546L). The fragments were ligated to Illumina sequencing adapters using the NEBNext Ultra II ligation module (E7595L) and size selected to 500 bp. The ligated target sequences were enriched with a KAPA library amplification kit (KK2621), again size selected to 500 bp, and normalized to 5 ng/μl using Qubit measurements. The library was sequenced on a partial lane of the Illumina HiSeq 2500 platform using 250-bp paired-end sequencing at the BYU DNA sequencing center.

In total, 1,780,201 paired-end reads passed Illumina default quality control filtering, representing an expected sequencing depth of 350× (6). To keep the expected nucleotide coverage below 200×, we assembled the genome from the first 1 million reads that passed the default Illumina quality filter using the shell split command. The reads were assembled using Velvet 1.2.10 (7) with automatic parameter optimization. We tested assemblies every 8 bp between 25mers and 250mers and then tested every 2 bp between 137mers and 165mers. The 157mer assembly minimized the number of contigs while maximizing the *N*_50_ value and maximum contig length. We submitted this assembly to RAST (8–10) and GenBank, where it was analyzed by the default annotation pipelines. The final annotated genome contained 3,511,265 bp, 139 contigs, a GC content of 44.2%, an *N*_50_ value of 118,894, and 3,339 coding sequences. The annotated genome was submitted to JSpeciesWS (11) in August 2020 for a tetra correlation search. Our isolate was most similar to *Lactiplantibacillus plantarum* strain WJL (coefficient, 0.99976). All software was used with default parameters unless otherwise noted.

Strain DmPark25_157 has two phage tail proteins, a murein hydrolase precursor, and a lipoyl transferase not found in *L. plantarum* WJL. Contig 62 of the DmPark25_157 assembly contains a *parA* chromosome partitioning gene and stress-resistant genes, suggesting that this contig could be a plasmid or horizontally acquired DNA involved in stress resistance.

### Data availability.

This whole-genome shotgun project has been deposited at DDBJ/ENA/GenBank under the accession number JACAOJ000000000; the raw reads have been deposited under the SRA accession number SRR13060588.
